# Gaussian Versus Mean-Field Models: Contradictory Predictions for the Casimir Force Under Dirichlet–Neumann Boundary Conditions

**DOI:** 10.3390/e27050468

**Published:** 2025-04-25

**Authors:** Daniel Dantchev, Vassil Vassilev, Joseph Rudnick

**Affiliations:** 1 Institute of Mechanics, Bulgarian Academy of Sciences, Academic Georgy Bonchev St. Building 4, 1113 Sofia, Bulgaria; vasilvas53@gmail.com; 2 Max-Planck-Institut für Intelligente Systeme, Heisenbergstrasse 3, D-70569 Stuttgart, Germany; 3 Department of Physics and Astronomy, University of California, Los Angeles, CA 90095, USA; jrudnickucla@gmail.com

**Keywords:** finite-size effects, exact results, Casimir force, mean-field model, Gaussian model, phase transitions, critical phenomena, phase diagrams

## Abstract

The mean-field model (MFM) is the workhorse of statistical mechanics: one normally accepts that it yields results which, despite differing numerically from correct ones, are not “very wrong”, in that they resemble the actual behavior of the system as eventually obtained by more advanced treatments. This, for example, turns out to be the case for the Casimir force under, say, Dirichlet–Dirichlet, (+,+) and (+,−) boundary conditions (BC) for which, according to the general expectations, the MFM is *attractive* for similar BC or *repulsive* for dissimilar BC force, with the principally correct position of the maximum strength of the force below or above the critical point $T_{c}$. It turns out, however, that this is *not* the case with Dirichlet–Neumann (DN) BC. In this case, the mean-field approach leads to an attractive Casimir force. This contradiction with the “boundary condition rule” is cured in the case of the Gaussian model under DN BC. Our results, which are mathematically exact, demonstrate that the Casimir force within the MFM is attractive as a function of temperature *T* and external magnetic field *h*, while for the Gaussian model, it is repulsive for h=0 and can be, surprisingly, both repulsive and attractive for h≠0. The treatment of the MFM is based on the exact solution of one non-homogeneous, nonlinear differential equation of second order. The Gaussian model is analyzed in terms of both its continuum and lattice realization. The obtained outcome teaches us that the mean-field results should be accepted with caution in the case of fluctuation-induced forces and ought to be checked against the more precise treatment of fluctuations within the envisaged system.

## 1. Introduction

Currently, the most prominent example of fluctuation-induced force is force due to quantum or thermal fluctuations of the electromagnetic field, leading to the so-called QED Casimir effect [1,2,3,4,5], named after the Dutch physicist H. B. Casimir who first realized that in the case of two perfectly-conducting, uncharged, and smooth plates parallel to each other in vacuum, at T=0, these fluctuations lead to an *attractive* force between them [1]. Nowadays, investigations devoted to that effect are performed on many fronts of research ranging from attempts to unify the four fundamental forces of nature [2,4,6] to rather practical issues such as the design and performance of MEMS and NEMS [7,8,9,10,11]. Let us explicitly mention the relation between the QED Casimir effect and cosmology [12]. Recent observations of the universe, such as the cosmological constant problem, underscore the importance of understanding vacuum forces at both microscopic and macroscopic scales. There has been speculation about possible relations between the Casimir effect and topics such as dark matter and cosmology [13,14,15,16,17]. These relations are linked to discussions about the physical meaning of the zero-point energy of quantum fields, the cosmological constant problem, and the physical interpretation of the Casimir effect. There is a considerable body of literature dealing with the physical source of the Casimir force. An extensive discussion of this issue can be found in [18,19] and more recently in [13,15,16,20,20]. It has been suggested [21,22] that hypothetical chameleon interactions, which might explain the mechanisms behind dark energy, might be detected through high-precision force measurements. In [22,23], the authors proposed the design, fabrication, and characterization of such a force sensor for the chameleon and Casimir force experiments using a parallel-plate configuration. The idea was to measure the total force between two parallel plates as a function of the density of a neutral gas allowed into the cavity. As the density of the gas increased, the mass of the chameleon field in the cavity increased, giving rise to a screening effect of the chameleon interaction.

Thirty years after Casimir, Fisher and De Gennes [24] showed that a very similar effect exists in critical fluids, known today as the critical Casimir effect. A summary of the results available for this effect can be found in recent reviews [25,26,27,28]. We note that the critical Casimir effect has been observed experimentally [29,30,31,32,33,34,35,36,37,38,39,40,41,42].

The description of the critical Casimir effect is based on the finite-size scaling theory [43,44,45,46]. Let us envisage a system with a film geometry $\infty^{d-1}\times L$, $L\equiv L_{⊥}$, and with boundary conditions ζ imposed along the spatial direction of finite extent *L*. Take $F_{\mathrm{tot}}^{\left(\zeta \right)}$ as the total free energy of such a system within the grand canonical ensemble (GCE). Then, if $f^{\left(\zeta \right)}\left(T,h,L\right)\equiv \lim_{A\to \infty }F_{\mathrm{tot}}^{\left(\zeta \right)}/A$ is the free energy per area *A* of the system, one can define the Casimir force for critical systems in the grand canonical (T−h)-ensemble (see [26,46,47,48]):

(1) $$\beta F_{\mathrm{Cas}}^{\left(\zeta \right)}\left(L,T,h\right)\equiv -\frac{\partial }{\partial L}f_{\mathrm{ex}}^{\left(\zeta \right)}\left(L,T,h\right),$$

where

(2) $$f_{\mathrm{ex}}^{\left(\zeta \right)}\left(L,T,h\right)\equiv f^{\left(\zeta \right)}\left(L,T,h\right)-Lf_{b}\left(T,h\right)$$

is the so-called excess (over the bulk) free energy per area and per $\beta^{-1}=k_{B}T$. Here, we suppose a system at temperature *T* is exposed to an external ordering field *h*, which couples linearly to its order parameter, such as the number density, the concentration difference, the magnetization, etc. Actually, the thermodynamic Casimir force $F_{\mathrm{Cas}}^{\left(\zeta \right)}\left(T,h,L\right)$ per area is the excess pressure over the bulk pressure due to the finite size (L<∞) of that system:

(3) $$F_{\mathrm{Cas}}^{\left(\zeta \right)}\left(T,h,L\right)=P_{L}^{\left(\zeta \right)}\left(T,h\right)-P_{b}\left(T,h\right).$$

Here, $P_{L}^{\left(\zeta \right)}$ is the pressure in the finite system under boundary condition ζ, while $P_{b}$ is the pressure in the infinite, i.e., macroscopically large, system. The above definition is actually equivalent to Equation (1). Note that $f_{\mathrm{ex}}^{\left(\zeta \right)}\left(L,T,h\right)$ is the excess grand potential per area, $f^{\left(\zeta \right)}\left(L,T,h\right)$ is the grand canonical potential per area of the finite system, and $f_{b}\left(T,h\right)$ is the grand potential per volume *V* for the macroscopically large system. The equivalence between the definitions in Equations (1) and (3) stems from the observation that for the finite system, one has $P_{L}=-\partial f^{\left(\zeta \right)}\left(L,T,h\right)/\partial L$, while for the bulk system, one has $f_{b}=-P_{b}$.

When $F_{\mathrm{Cas}}^{\left(\zeta \right)}\left(L,t,h\right)<0$, the excess pressure is inward, i.e., towards the system. This means there is *attraction* between surfaces of the system towards each other and *repulsion* if $F_{\mathrm{Cas}}^{\left(\zeta \right)}\left(L,t,h\right)>0$.

In the remainder of this article, we will consider the behavior of the Casimir force under periodic and Neumann–Dirichlet boundary conditions within the Gaussian and mean-field models. These are two of the principal models of statistical physics. We will show that they might produce contradictory predictions for the behavior of the Casimir force, including whether the force for given *T* and *h* is attractive or repulsive. Before continuing to the specific calculations used, let us mention the Gaussian model has been intensively used to study the behavior of the critical Casimir effect [46,49,50,51,52,53,54,55], as well as the Ising mean-field model [53,56,57,58,59,60,61,62,63,64] (for a review, see [26]).

We start by considering the behavior of the Casimir force within the Gaussian model, both for its continuum and lattice versions.

## 2. The Casimir Force Within the Continuum Gaussian Model

The continuum version of the Gaussian model with a scalar order parameter consists of linear and bilinear terms in the Ginzburg–Landau–Wilson formulation of a system in *d* dimensions that undergoes a continuous symmetry-breaking phase transition at low temperatures. The partition function of this system is the functional integral,

(4) $${Ƶ}_{G}\left(t,h\right)=\int \exp\left[-F\left(\psi \left(\vec{r}\right)\right)\right]\;D\left\{\psi \left(\vec{r}\right)\right\},$$

where

(5) $$F\left(\psi \left(\vec{r}\right)\right)=\int \left[t\psi {\left(\vec{r}\right)}^{2}+{\left|\vec{\nabla }\psi \left(\vec{r}\right)\right|}^{2}-h\psi \left(\vec{r}\right)\right]\;d^{d}r.$$

In (5), *t* is the reduced temperature proportional to $T-T_{c}$, and *h* is the spatially constant ordering field. Because of the Gaussian nature of the free energy functional $F(\psi \left(\vec{r}\right))$, the partition function resolves into the following product:

(6) $${Ƶ}_{G}\left(t,h\right)={Ƶ}_{G,I}\left(t\right)\times {Ƶ}_{G,h}\left(t,h\right),$$

where ${Ƶ}_{G,I}\left(t\right)$ is the partition function of the system with h=0. The geometry of the system under consideration is a slab of a large, ultimately infinite, cross section and finite thickness *L*.

With regard to scaling considerations, there are two combinations of parameters that reflect the predictions of finite size scaling. They are as follows:

(7) $$\begin{array}{ccc} x_{t} & = & tL^{1/\nu }=tL^{2}, \end{array}$$

(8) $$\begin{array}{ccc} x_{h} & = & hL^{(d+2-\eta )/2}=hL^{(d+2)/2}, \end{array}$$

where ν is the correlation length exponent and is equal to 1/2 in the Gaussian model, and, as noted above, *d* is the dimensionality of the system. Our end results for the Casimir forces acting upon the systems will depend on the boundary conditions imposed. In all cases, the form of the Casimir force is as follows:

(9) $$\begin{array}{ccc} f_{\mathrm{Cas}}\left(t,h,L\right) & = & L^{-d}\left(w_{\mathrm{Cas},I}\left(x_{t}\right)+x_{h}^{2}w_{\mathrm{Cas},h}\left(x_{t}\right)\right). \end{array}$$

All results reported in this portion of the article rely on two results, which can be obtained with the use of contour integration techniques (see also [65]). The two results are as follows:

(10) $$\begin{array}{ccc} \sum_{n=-\infty }^{\infty }\frac{1}{an^{2}+b} & = & \frac{\pi \coth(\pi \sqrt{b/a})}{\sqrt{ab}}, \end{array}$$

(11) $$\begin{array}{ccc} \sum_{n=0}^{\infty }\frac{1}{c{\left(2n+1\right)}^{2}+d} & = & \frac{\pi \tanh(\pi /2\sqrt{d/c})}{4\sqrt{cd}}. \end{array}$$

In order to carry out the evaluation of the free energy of the Gaussian model, we turn to the basic set of functions that will be used to construct the free energy with and without an ordering field. These functions allow us to evaluate the partition function by integrating the amplitudes of the contributions of each member of the set to the order parameter. Here, we focus on periodic boundary conditions. Ignoring the dependence on position in the “plane” of the slab, the functions include the orthonormal set:

(12) $$\begin{array}{ccc} \psi_{c}^{\left(n\right)}\left(z\right) & = & \sqrt{2/L}\cos\left(2\pi nz/L\right), \end{array}$$

(13) $$\begin{array}{ccc} \psi_{s}^{\left(n\right)}\left(z\right) & = & \sqrt{2/L}\sin\left(2\pi nz/L\right), \end{array}$$

(14) $$\begin{array}{ccc} \psi_{0}\left(z\right) & = & \sqrt{1/L}, \end{array}$$

with *n* as a positive integer. It is straightforward to show that this set is orthonormal as a function of *z* in that

(15) $$\begin{array}{ccc} \int_{0}^{L}\psi_{c}^{\left(n\right)}\left(z\right)\psi_{c}^{\left(m\right)}\left(z\right)\;dz & = & \delta_{m,n}, \end{array}$$

(16) $$\begin{array}{ccc} \int_{0}^{L}\psi_{s}^{\left(n\right)}\left(z\right)\psi_{s}^{\left(m\right)}\left(z\right)\;dz & = & \delta_{m,n}, \end{array}$$

(17) $$\begin{array}{ccc} \int_{0}^{L}\psi_{0}{\left(z\right)}^{2}\;dz & = & 1. \end{array}$$

The three function types are all mutually orthogonal. In the case of higher dimensions, we construct a new basic set by multiplying functions (12)–(14) by suitable functions of the orthogonal position variables. Those functions can be taken to be of the form $e^{i\vec{Q}\cdot \vec{R}}$, where $\vec{R}$ is a d−1-dimensional position vector in the plane of the slab, and $\vec{Q}$ is in its reciprocal space.

We then express the order parameter as follows:

(18) $$\psi \left(z,\vec{R}\right)=\sum_{\vec{Q}}e^{i\vec{Q}\cdot \vec{R}}\left(\sum_{n=1}^{\infty }a_{n}^{\left(c\right)}\psi_{c}^{\left(n\right)}\left(z\right)+\sum_{n=1}^{\infty }a_{n}^{\left(s\right)}\psi_{c}^{\left(n\right)}\left(z\right)+a_{0}\psi_{0}\left(z\right)\right).$$

The free energy for a given configuration of the Gaussian order parameter, in terms of the amplitudes in the expansion of the order parameter in the basis set (15)–(17), is as follows:

(19) $$\sum_{\vec{Q}}\left[\sum_{n=1}^{\infty }\left(a_{n}^{(c)\;2}+a_{n}^{(s)\;2}\right)\left(t+Q^{2}+{\left(2\pi n/L\right)}^{2}\right)+a_{0}^{2}t-ha_{0}\sqrt{L}\right].$$

The last term in the brackets above reflects the fact that only the basis function that the constant external field couples to is the constant function in (14).

The next step is to exponentiate the expression in (19), multiply either −1/β or setting β=1 by −1, and, after that, perform the Gaussian integrals over the $a_{n}^{\left(c\right)}$’s, the $a_{n}^{\left(s\right)}$’s, and $a_{0}$. The resulting partition function is provided as follows:

(20) $$Ƶ=\exp\left[\frac{1}{\beta }\left(\frac{h^{2}LA}{4t}+\frac{A}{{\left(2\pi \right)}^{d-1}}\int \;d^{d-1}Q\sum_{n=-\infty }^{\infty }\frac{1}{2}\ln\left(\frac{t+Q^{2}+{\left(2\pi n/L\right)}^{2}}{\pi }\right)\right)\right].$$

The coefficient *A* in (20) is the d−1 dimensional area of the slab.

As our next step, we evaluate the sum over *n* on the right-hand side of the expression for the partition function. To achieve this, we take the *t*-derivative of the logarithm of the summand, perform the sum over *n*, and then integrate the resulting expression with respect to *t*. Taking the derivative of the summand in (20) with respect to *t* leaves us with the following sum:

(21) $$\begin{array}{c} \frac{1}{2}\sum_{n=-\infty }^{\infty }\frac{1}{t+Q^{2}+{\left(2\pi n/L\right)}^{2}}=\frac{L\coth\left(\frac{1}{2}L\sqrt{Q^{2}+t}\right)}{2\sqrt{Q^{2}+t}}, \end{array}$$

which follows from (10). This integrates up to the following:

(22) $$\begin{array}{c} 2\ln\left(\sinh\left(L\sqrt{Q^{2}+t}/2\right)\right). \end{array}$$

The large *L* limit of (22) is as follows:

(23) $$L\sqrt{t+Q^{2}}.$$

To determine the contribution to the Casimir force per unit area, we take the *L* derivative of the difference between (23) and (22) and then integrate over $\vec{Q}$. The derivative yields the following:

(24) $$\frac{1}{2}\sqrt{Q^{2}+t}\left(1-\coth(L\sqrt{Q^{2}+t})\right)=-\sqrt{Q^{2}+t}\frac{e^{-L\sqrt{Q^{2}+t}}}{e^{L\sqrt{Q^{2}+t}}-e^{-L\sqrt{Q^{2}+t}}}.$$

The sum over values of $\vec{Q}$ is expressible as an integral, which takes the following form:

(25) $$\begin{array}{cc} - & \frac{K_{d-1}}{{\left(2\pi \right)}^{d-1}}\int_{0}^{\infty }Q^{d-2}\sqrt{Q^{2}+t}\frac{e^{-L\sqrt{Q^{2}+t}}}{e^{L\sqrt{Q^{2}+t}}-e^{-L\sqrt{Q^{2}+t}}}\;dQ\\ & =\frac{1}{L^{d}}\left(-\frac{K_{d-1}}{{\left(2\pi \right)}^{d-1}}x_{t}^{d/2}\int_{0}^{\infty }w^{d-2}\sqrt{1+w^{2}}\frac{e^{-\sqrt{x_{t}\left(1+w^{2}\right)}}}{e^{\sqrt{x_{t}\left(1+w^{2}\right)}}-e^{-\sqrt{x_{t}\left(1+w^{2}\right)}}}\;dw\right)\\ & =\frac{1}{L^{d}}X_{I}^{\left(\mathrm{per},3\right)}\left(x_{t}\right), \end{array}$$

where, to obtain the last line of (25), we define a new integration variable $w=Q/\sqrt{t}$ and then make use of definition (7) of $x_{t}$. The implication of (25) is that we can express the h=0 contribution to the Casimir force as $L^{-d}$ times a function of the scaling temperature variable $x_{t}$. The coefficient $K_{d}$ in the equations above is the geometric factor:

(26) $$K_{d}=\frac{2\pi^{d/2}}{\Gamma \left(\frac{d}{2}\right)}.$$

**Figure 1:**
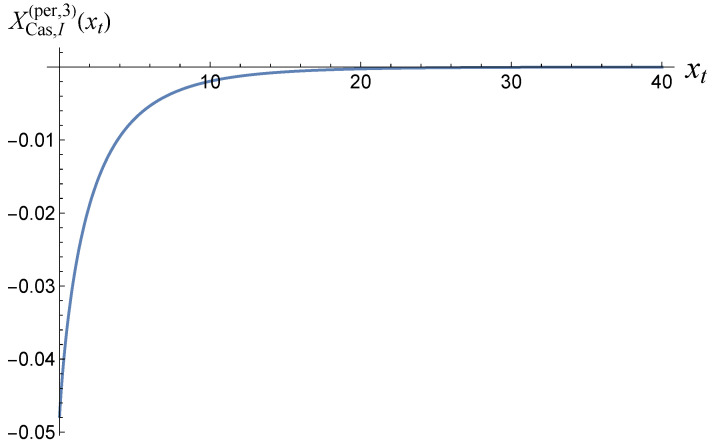
The function $X_{\mathrm{Cas},I}^{\mathrm{per},3}\left(x_{t}\right)$ plotted versus $x_{t}$.

In the case of three dimensions, further processing of the result (25) is possible. We determine the following:

(27) $$X_{\mathrm{Cas},I}^{\mathrm{per},3}\left(x_{t}\right)=-\frac{2\sqrt{x_{t}}{\mathrm{Li}}_{2}\left(e^{-2\sqrt{x_{t}}}\right)+{\mathrm{Li}}_{3}\left(e^{-2\sqrt{x_{t}}}\right)-2x_{t}\log\left(1-e^{-2\sqrt{x_{t}}}\right)}{8\pi },$$

where ${\mathrm{Li}}_{j}\left(x\right)$ is the polylogarithm function (see [66]). A plot of the function $X_{\mathrm{Cas},I}^{\mathrm{per},3}\left(x_{t}\right)$ is shown in Figure 1.

The first term in parentheses in Equation (20) provides us with the *h*-dependent contribution to the free energy: $-h^{2}LA/4t$. This is to be compared to the corresponding free energy of a neighboring bulk phase, which is $-h^{2}\left(L_{0}-L\right)A/4t$, where $L_{0}$ is an extent that will ultimately be taken to infinity. If you add two free energies, the dependence on *L*, i.e., the thickness of the slab, disappears. This means that there is no *h*-dependent free energy when slab boundary conditions are periodic; hence, there is no *h*-dependent contribution to the Casimir force.

The calculations in the case of periodic boundary conditions point the way to evaluating the partition function and Casimir force of the Dirichlet–Neumann boundary conditions.

In this case, (unnormalized) basis functions are exclusive of their dependence on in-plane coordinates:

(28) sin((2n+1)πz/2L).

**Figure 2:**
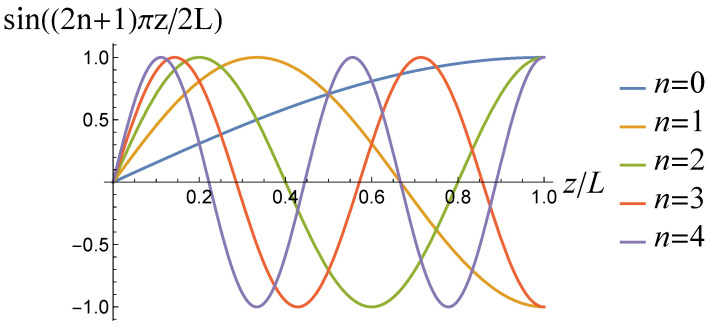
The functions in (28).

Examples of these functions are shown in Figure 2.

Focusing on the *h*-independent contribution to the partition function, the sum to perform in this case is as follows (see (11)):

(29) $$\frac{1}{2}\sum_{n=0}^{\infty }\frac{1}{t+Q^{2}+{\left(\left(2n+1\right)\pi /2L\right)}^{2}}=\frac{L\tanh\left(L\sqrt{t+Q^{2}}\right)}{4\sqrt{t+Q^{2}}}.$$

Note that in the large limit *L*, the right-hand side transitions to the expected asymptotic form. If we subtract that limiting form and integrate it with respect to *t*, we are left with the following:

(30) $$\frac{1}{2}\left(\log\left(\cosh\left(L\sqrt{t+Q^{2}}\right)\right)-L\sqrt{t+Q^{2}}\right).$$

Finally, we subtract the derivative of this with respect to *L*, leaving us with the following:

(31) $$\frac{1}{2}\left(-\sqrt{r+Q^{2}}\tanh\left(L\sqrt{t+Q^{2}}\right)+\sqrt{t+Q^{2}}\right)=\sqrt{t+Q^{2}}\frac{e^{-L\sqrt{t+Q^{2}}}}{e^{L\sqrt{t+Q^{2}}}+e^{-L\sqrt{t+Q^{2}}}}.$$

Making use of the analysis of previous sections, this leaves us with the following result for the Casimir force in the case of the *d*-dimensional Gaussian model with Dirichlet–Neumann boundary conditions:

(32) $$\begin{array}{c} \frac{K_{d-1}}{{\left(2\pi \right)}^{d-1}}\int_{0}^{\infty }Q^{d-2}\sqrt{t+Q^{2}}\frac{e^{-L\sqrt{t+Q^{2}}}}{e^{L\sqrt{t+Q^{2}}}+e^{-L\sqrt{t+Q^{2}}}}dQ\\ \;=\frac{K_{d-1}}{{\left(2\pi \right)}^{d-1}}\frac{1}{L^{d}}{\left(x_{t}\right)}^{d/2}\int_{0}^{\infty }w^{d-2}\sqrt{1+w^{2}}\frac{e^{-\sqrt{x_{t}}\sqrt{1+w^{2}}}}{e^{\sqrt{x_{t}}\sqrt{1+w^{2}}}+e^{-\sqrt{x_{t}}\sqrt{1+w^{2}}}}dw\\ \;=\frac{1}{L^{d}}X_{\mathrm{Cas},D/N}^{\left(d\right)}\left(x_{t}\right). \end{array}$$

When d=3, we have the following:

(33) $$X_{\mathrm{Cas},D/N,I}^{\left(3\right)}\left(x_{t}\right)=-\frac{2\sqrt{x_{t}}{\mathrm{Li}}_{2}\left(-e^{-2\sqrt{x_{t}}}\right)+{\mathrm{Li}}_{3}\left(-e^{-2\sqrt{x_{t}}}\right)-2x_{t}\log\left(e^{-2\sqrt{x_{t}}}+1\right)}{8\pi }.$$

**Figure 3:**
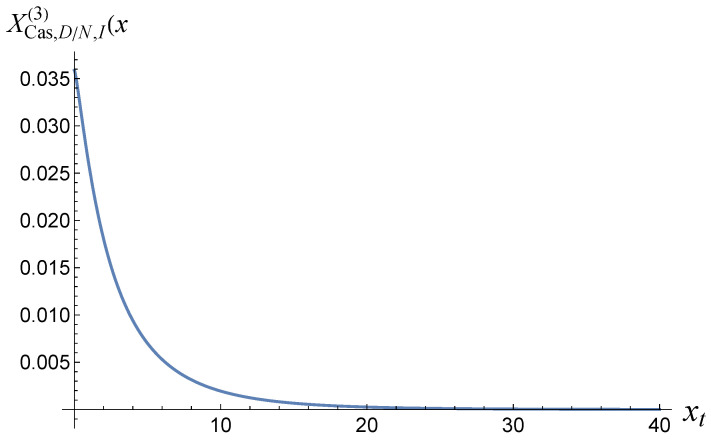
The function $X_{\mathrm{Cas},D/N}^{\left(3\right)}\left(x_{t}\right)$, as provided in (33).

Figure 3 shows what the function $X_{\mathrm{Cas},D/N,I}^{\left(d\right)}\left(x_{t}\right)$ looks like when d=3.

In order to determine the *h*-dependent contribution to the Casimir force, we turn to the normalized basic set in the case of Dirichlet–Neumann boundary conditions. Assuming that the boundary conditions are Dirichlet at z=0 and Neumann at z=L, this basis set is as follows:

(34) $$\psi_{DN}^{\left(n\right)}\left(z\right)=\sqrt{2/L}\sin\left((n+1/2)\pi z/L\right),$$

with *n* as an integer and

(35) 0≤n<∞.

It is straightforward to establish the following:

(36) $$\int_{0}^{L}\psi_{DN}^{\left(n\right)}{\left(z\right)}^{2}\;dL=1,$$

while

(37) $$\int_{0}^{L}\psi_{DN}^{\left(n\right)}\left(z\right)\;dL=\frac{2\sqrt{2L}}{(2n+1)\cdot \pi }$$

As it turns out, there is no need to take into account any dependence of the basic set on coordinates in the plane of the slab. This is because a constant ordering field couples only to order parameter configurations that are independent of those coordinates.

With this in mind, we expand the order parameter as follows:

(38) $$\Psi \left(z\right)=\sum_{n=0}^{\infty }a_{n}^{\left(DN\right)}\psi_{DN}^{\left(n\right)}\left(z\right).$$

The Gaussian integrations over $a_{n}^{\left(DN\right)}$’s leaves us with the summation over *n* for the *h*-dependent contribution to the partition function:

(39) $$\begin{array}{c} \exp\left[h^{2}\sum_{n=0}^{\infty }{\left(\frac{2\sqrt{2L}}{(2n+1)\pi }\right)}^{2}\frac{1}{4({\left(\pi \left(n+1/2\right)/L\right)}^{2}+t)}\right]\\ \;\;=\exp\left[h^{2}\left(\frac{L}{4t}-\frac{\tanh(L\sqrt{t})}{4t^{3/2}}\right)\right]\\ \;\;=\exp\left[\frac{h^{2}}{4t^{3/2}}\left(L\sqrt{t}-\tanh\left(L\sqrt{t}\right)\right)\right], \end{array}$$

where the evaluation of the sum over *n* in (39) is accomplished with the use of (11) and a partial fraction decomposition of the summand. The first term in parentheses on the last line of (39) provides exactly the same expression as the *h*-dependent contribution to the partition function of the slab with periodic boundary conditions. Its influence on the Casimir force is exactly canceled by the influence of the bulk. The following remains:

(40) $$\begin{array}{ccc} -h^{2}\frac{\partial }{\partial L}\tanh\left(L\sqrt{t}\right)/4t^{3/2} & = & -\frac{h^{2}}{4t}{\mathrm{sech}}^{2}\left(L\sqrt{t}\right)\\ & = & -\frac{h^{2}L^{2}}{4x_{t}}{\mathrm{sech}}^{2}\left(\sqrt{x_{t}}\right)\\ & = & -\frac{1}{L^{d}}\frac{x_{h}^{2}}{4x_{t}}{\mathrm{sech}}^{2}\left(\sqrt{x_{t}}\right), \end{array}$$

where we make use of the definition of the scaling combination $x_{h}$ in (8). The scaling form of the contribution to the Casimir force is then as follows:

(41) $$X_{D/N}^{\left(3\right)}\left(x_{t},x_{h}\right)=\frac{-x_{h}^{2}}{4x_{t}}\overset{2}{\mathrm{sech}}\left(\sqrt{x_{t}}\right).$$

**Figure 4:**
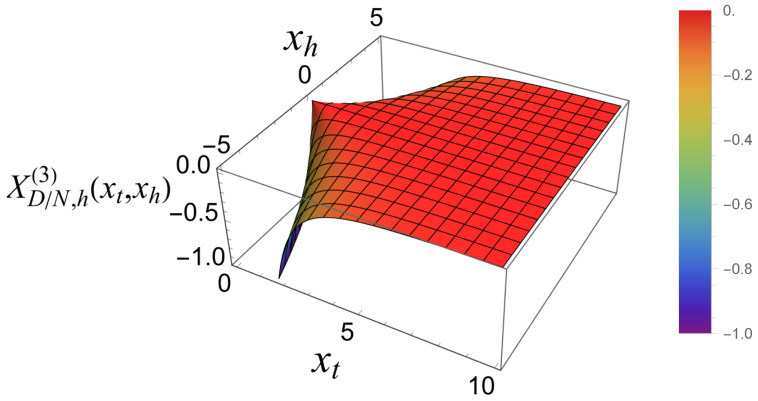
The function $X_{D/N,h}^{\left(3\right)}\left(x_{t},x_{h}\right)$, as presented by (41).

This function is shown in Figure 4. Note that this function is *always attractive*.

The total scaling function $X_{D/N}^{\left(3\right)}\left(x_{t},x_{h}\right)$ is provided by the following:

(42) $$X_{D/N,h}^{\left(3\right)}\left(x_{t},x_{h}\right)=-\frac{2\sqrt{x_{t}}{\mathrm{Li}}_{2}\left(-e^{-2\sqrt{x_{t}}}\right)+{\mathrm{Li}}_{3}\left(-e^{-2\sqrt{x_{t}}}\right)-2x_{t}\log\left(e^{-2\sqrt{x_{t}}}+1\right)}{8\pi }-\frac{x_{h}^{2}}{4x_{t}}{\mathrm{sech}}^{2}\left(\sqrt{x_{t}}\right).$$

**Figure 5:**
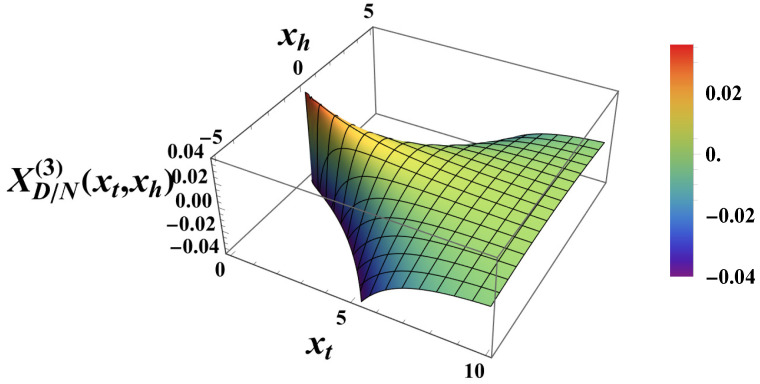
The total scaling contribution to the Casimir force for Dirichlet–Neumann boundary conditions in the three dimensional Gaussian model with a scalar order parameter, $X_{D/N}^{\left(3\right)}\left(x_{t},x_{h}\right)$. Note that this function can be both positive (repulsive) and negative (attractive).

Figure 5 shows what this function looks like.

**Figure 6:**
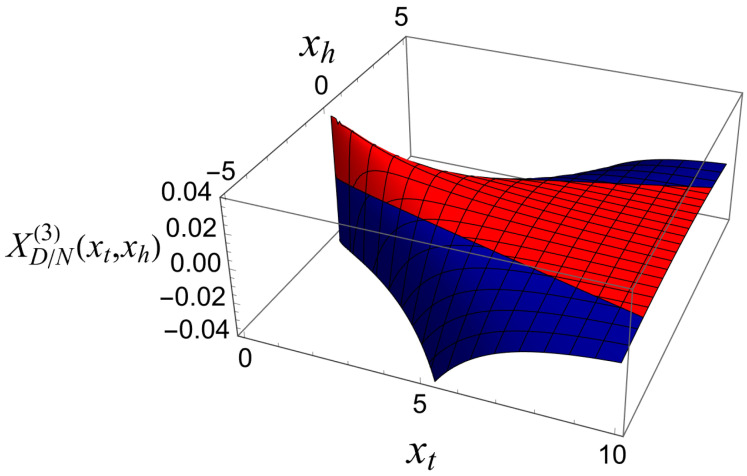
The total scaling contribution to the Casimir force for Dirichlet–Neumann boundary conditions in the three-dimensional Gaussian model with a scalar order parameter, $X_{D/N}^{\left(3\right)}\left(x_{t},x_{h}\right)$. The red region in the figure corresponds to a repulsive force, and the blue region corresponds to an attractive force.

Another depiction of the scaling contribution to the Casimir force for Dirichlet–Neumann boundary conditions in the three-dimensional Gaussian model with a scalar order parameter, $X_{D/N}^{\left(3\right)}\left(x_{t},x_{h}\right)$, is shown in Figure 6. The figure highlights the regions in which the function is attractive and repulsive.

## 3. The Casimir Force Within the Lattice Gaussian Model

We consider a ferromagnetic model with nearest-neighbor interactions on a fully finite *d*-dimensional hypercubic lattice $\Lambda \in Z^{d}$ of |Λ| sites. Let us take $\Lambda \in Z^{d}$ to be the parallelepiped $\Lambda =L_{1}\times \cdots \times L_{d}$, where × denotes the direct (Cartesian) product of the finite sets $L_{\nu }=\left\{1,∆,L_{\nu }\right\}$.

It is convenient to consider the configuration space $\Omega_{\Lambda }=R^{\left|\Lambda \right|}$ as a Euclidean vector space in which each configuration is represented by a column vector $S_{\Lambda }$ with components labeled according to the lexicographic order of the set $\left\{r=\left(r_{1},\cdots ,r_{d}\right)\in \Lambda \right\}$. Let $S_{\Lambda }^{†}$ be the corresponding transposed row vector, and let the dot (·) denote matrix multiplication. Then, the given boundary conditions $\tau =(\tau_{1},\cdots ,\tau_{d})$, specified for each pair of opposite faces of Λ by some $\tau_{\nu }$, take the following form:

(43) $$\beta H_{\Lambda }^{\left(\tau \right)}\left(S_{\Lambda }|K\right)=-\frac{1}{2}KS_{\Lambda }^{†}\cdot Q_{\Lambda }^{\left(\tau \right)}\cdot S_{\Lambda }.$$

Here, K=βJ, where *J* is the interaction constant (to be set to J=1 in the remainder), and the |Λ|×|Λ| interaction matrix $Q_{\Lambda }^{\left(\tau \right)}$ can be written as follows:

(44) $$Q_{\Lambda }^{\left(\tau \right)}=\left(\Delta_{1}^{\left(\tau_{1}\right)}+2\;E_{1}\right)\times \cdots \times \left(\Delta_{d}^{\left(\tau_{d}\right)}+2\;E_{d}\right),$$

where $\Delta_{\nu }^{\left(\tau_{\nu }\right)}$ is the one-dimensional discrete Laplacian defined on the finite chain $L_{\nu }$ under boundary condition $\tau_{\nu }$, and $E_{\nu }$ is the $L_{\nu }\times L_{\nu }$ unit matrix.

By using the results of ([46], Chapter 7), we can write down the eigenfunctions of the interaction matrix (44) in the following form:

(45) $$u_{\Lambda }^{\left(\tau \right)}\left(r,k\right)=u_{L_{1}}^{\left(\tau_{1}\right)}\left(r_{1},k_{1}\right)\;\cdots \;u_{L_{d}}^{\left(\tau_{d}\right)}\left(r_{d},k_{d}\right),\;k=\left(k_{1},\cdots ,k_{d}\right)\in \Lambda ,$$

Then, we can obtain the corresponding eigenvalues:

(46) $$\mu_{\Lambda }^{\left(\tau \right)}\left(k\right)=2\sum_{\nu =1}^{d}\cos\varphi_{L_{\nu }}^{\left(\tau_{\nu }\right)}\left(k_{\nu }\right),\;k\in \Lambda .$$

Obviously, $\max_{k\in \Lambda }\mu_{\Lambda }^{\left(\tau \right)}\left(k\right)=2d$. Note that the interaction Hamiltonian (43) has negative eigenvalues, which makes the inclusion of a positive definite quadratic form in the Gibbs exponent necessary to ensure the existence of the corresponding partition function. Thus, we consider the following Hamiltonian:

(47) $$\beta H_{\Lambda }^{\left(\tau \right)}\left(S_{\Lambda }|\beta ,h_{\Lambda };s\right)=-\frac{1}{2}\beta S_{\Lambda }^{†}\cdot Q_{\Lambda }^{\left(\tau \right)}\cdot S_{\Lambda }+s\;S_{\Lambda }^{†}\cdot S_{\Lambda }-h_{\Lambda }^{†}\cdot S_{\Lambda }.$$

Here, $h_{\Lambda }=\left\{h\left(r\right),\;r\in \Lambda \right\}$ is a column vector representing (in units of $k_{B}T$) the inhomogeneous magnetic field configuration acting upon the system, indicating $h_{\Lambda }^{†}$ is the transposed row-vector.

In order to ensure the existence of the partition function, all the eigenvalues $-\frac{1}{2}\beta \mu_{\Lambda }^{\left(\tau \right)}\left(k\right)+s$k∈Λ of the quadratic form in $\beta H_{\Lambda }^{\left(\tau \right)}\left(S_{\Lambda }|\beta ,h_{\Lambda };s\right)$ ought to be positive. Hence, the field $s^{\left(\tau \right)}$ must satisfy the following inequality:

(48) $$s>\frac{1}{2}\beta \max_{k\in \Lambda }\mu_{\Lambda }^{\left(\tau \right)}\left(k\right)\equiv \frac{1}{2}\beta \mu_{\Lambda }^{\left(\tau \right)}\left(k_{0}\right),$$

with

(49) $$\beta_{c,L}=\frac{1}{2}\mu_{\Lambda }^{\left(\tau \right)}\left(k_{0}\right)$$

defining the critical temperature of the *finite* system. Since, as stated above, $\max_{k\in \Lambda }\mu_{\Lambda }^{\left(\tau \right)}\left(k\right)=2d$, it is clear that for the infinite system,

(50) $$\beta_{c}=d.$$

The free energy density of a *finite* system in Λ region is as follows:

(51) $$\begin{array}{c} \beta f_{\Lambda }^{\left(\tau \right)}\left(\beta ,h_{\Lambda }\right)=\frac{1}{2}\left\{\ln\left(\beta /2\pi \right)-2s+\;U_{\Lambda }^{\left(\tau \right)}\left(\beta ,s\right)-P_{\Lambda }^{\left(\tau \right)}\left(\beta ,h_{\Lambda },s\right)\right\}. \end{array}$$

In Equation (51), the first two terms do not depend on the size of the system, i.e., they are the same in both finite and infinite systems. The other two terms do depend, however, on the size of the system. The function $U_{\Lambda }^{\left(\tau \right)}\left(\beta ,s\right)$ is due to the spin–spin interaction (and will be called “interaction term”); it depends on *s*, but does not depend on *h*. It is equal to the following:

(52) $$U_{\Lambda }^{\left(\tau \right)}\left(\beta ,s\right)={\left|\Lambda \right|}^{-1}\sum_{k\in \Lambda }\ln\left[\frac{2s}{\beta }-\mu_{\Lambda }^{\left(\tau \right)}\left(k\right)\right],$$

and it is obtained after performing the corresponding Gaussian integrals in the free energy of the finite system. The dependence of the free energy on the field variables *h* is provided by the “field term”:

(53) $$P_{\Lambda }^{\left(\tau \right)}\left(\beta ,h_{\Lambda };s\right)=\frac{1}{\beta |\Lambda |}\sum_{k\in \Lambda }\frac{|{\hat{h}}_{\Lambda }^{\left(\tau \right)}{\left(k\right)|}^{2}}{2s/\beta -\mu_{\Lambda }^{\left(\tau \right)}\left(k\right)}.$$

Here, ${\hat{h}}_{\Lambda }^{\left(\tau \right)}\left(k\right)$ denotes the projection of the magnetic field configuration $h_{\Lambda }$ on the eigenfunction $\{{\bar{u}}_{\Lambda }^{\left(\tau \right)}\left(r,k\right),$k∈Λ} (by $\bar{u}$, we denote the complex conjugate of u∈C):

(54) $${\hat{h}}_{\Lambda }^{\left(\tau \right)}\left(k\right)=\sum_{r\in \Lambda }h\left(r\right){\bar{u}}_{\Lambda }^{\left(\tau \right)}\left(r,k\right).$$

Defining $\beta_{c}$, the following is true:

(55) $$\frac{2s}{\beta }=2d\frac{\beta_{c}}{\beta },$$

and the above expressions can be rewritten as follows:

(56) $$U_{\Lambda }^{\left(\tau \right)}\left(\beta \right)={\left|\Lambda \right|}^{-1}\sum_{k\in \Lambda }\ln\left[2d\left(\beta_{c}/\beta -1\right)+2d-\mu_{\Lambda }^{\left(\tau \right)}\left(k\right)\right],$$

Then,

(57) $$P_{\Lambda }^{\left(\tau \right)}\left(\beta ,h_{\Lambda }\right)=\frac{1}{\beta |\Lambda |}\sum_{k\in \Lambda }\frac{|{\hat{h}}_{\Lambda }^{\left(\tau \right)}{\left(k\right)|}^{2}}{2d\left(\beta_{c}/\beta -1\right)+2d-\mu_{\Lambda }^{\left(\tau \right)}\left(k\right)}.$$

Using the notations of ([46], Chapter 7), we provide a list of the complete sets of orthonormal eigenfunctions, $\{u_{L}^{\left(\tau \right)}\left(r,k\right)$ and k=1,⋯,L}, of the one-dimensional discrete Laplacian under the Neumann–Dirichlet (ND) boundary conditions:

- Periodic (p) boundary conditions:
  (58) $$u_{L}^{\left(p\right)}\left(r,k\right)=L^{-1/2}\exp\left[-ir\varphi_{L}^{\left(p\right)}\left(k\right)\right].$$
- Neumann–Dirichlet (ND) boundary conditions:
  (59) $$u_{L}^{\left(\mathrm{ND}\right)}\left(r,k\right)=2{\left(2L+1\right)}^{-1/2}\cos\left(r-1/2\right)\varphi_{L}^{\left(\mathrm{ND}\right)}\left(k\right).$$

The quantities $\varphi_{L}^{\left(\tau \right)}$ and k=1,∆,L are defined as follows:

(60) $$\begin{array}{cc} \varphi_{L}^{\left(p\right)}\left(k\right)=2\pi k/L,\; & \varphi_{L}^{\left(\mathrm{ND}\right)}\left(k\right)=\pi \left(2k-1\right)/\left(2L+1\right). \end{array}$$

Now, we are ready to determine the finite-size behavior of the Gaussian model under the Dirichlet–Neumann boundary conditions. According to Equation (59), S(0)=S(1), i.e., one has the realization of Neumann boundary conditions, while L+1=0, which corresponds to Dirichlet boundary conditions. Thus, in the envisaged one-dimensional chain, one has *L* independent spin variables $\left\{S(1),S(2),\cdots ,S(L)\right\}$.

We start with the consideration of a d=3 dimensional system:

- Under fully periodic (p) boundary conditions, i.e., τ=(p,p,p), one has $k_{0}=\left(L_{1},L_{2},L_{3}\right)$; hence, $\mu_{\Lambda }^{\left(p,p,p\right)}\left(k_{0}\right)=6$.
- Under Neumann–Dirichlet boundary conditions along the *z* direction, i.e., τ=(p,p,ND), one has $k_{0}=\left(L_{1},L_{2},1\right)$; hence, $\mu_{\Lambda }^{\left(p,p,\mathrm{ND}\right)}\left(k_{0}\right)=4+2\cos\left[\pi /\left(2L+1\right)\right]$.

### 3.1. The Gaussian Model on a Lattice for the d=3 Case

We recall that for this model, α=1/2,γ=1 and ν=1/2 [46,67].

#### 3.1.1. TheBehavior of the Interaction Term $U_{\Lambda }^{\left(\tau \right)}\left(\beta \right)$

We set τ=(p,p,ND) and use the short-hand notation τ=ND for these boundary conditions. Then, we perform Equation (52) in the limits $L_{1},L_{2}\to \infty$, keeping $L_{3}=L$ fixed. For the interaction term, one then obtains the following:

(61) $$U_{L,3}^{\left(\mathrm{ND}\right)}\left(\beta \right)=\lim_{L_{1},L_{2}\to \infty }U_{\Lambda }^{\left(p,p,\mathrm{ND}\right)}\left(\beta \right)=\frac{1}{L}\sum_{k=1}^{L}V_{2}\left[6\left(\beta_{c}/\beta -1\right)+2\left(1-\cos\pi \frac{2k-1}{2L+1}\right)\right],$$

where

(62) $$V_{d}\left(z\right):=\frac{1}{{\left(2\pi \right)}^{d}}\int_{-\pi }^{\pi }d\theta_{1}\cdots \int_{-\pi }^{\pi }d\theta_{d}\ln\left[z+2\sum_{\nu =1}^{d}\left(1-\cos\theta_{\nu }\right)\right].$$

#### 3.1.2. The Behavior of the Interaction Term in the Bulk System

In accordance with Equation (62), one obtains the following:

(63) $$U_{\infty ,3}\left(\beta \right)=V_{3}\left[6(\beta_{c}/\beta -1)\right].$$

#### 3.1.3. The Behavior of the Interaction Term in the Film System with Neumann–Dirichlet Boundary Conditions

Explicitly, from Equation (61), one obtains the following:

(64) $$U_{L,3}^{\left(\mathrm{ND}\right)}\left(\beta \right)=\frac{1}{L}\sum_{k=1}^{L}V_{2}\left[6\left(\beta_{c}/\beta -1\right)+2\left(1-\cos\pi \frac{2k-1}{2L+1}\right)\right]=\frac{1}{{\left(2\pi \right)}^{2}}\;\int_{-\pi }^{\pi }d\theta_{1}\int_{-\pi }^{\pi }d\theta_{2}\;S^{\left(\mathrm{ND}\right)}\left(\beta ,L|\theta_{1},\theta_{2}\right),$$

with

(65) $$S^{\left(\mathrm{ND}\right)}\left(\beta ,L|\theta_{1},\theta_{2}\right)=\frac{1}{L}\sum_{k=1}^{L}\;\ln\left[6\left(\beta_{c}/\beta -1\right)+2\sum_{\nu =1}^{2}\left(1-\cos\theta_{\nu }\right)+2\left(1-\cos\pi \frac{2k-1}{2L+1}\right)\right].$$

This sum is of the following form:

(66) $$S^{\left(\mathrm{ND}\right)}\left(x,L\right)=\frac{1}{L}\ln\prod_{k=0}^{L-1}\;2\left[\cosh\left(x\right)-\cos\pi \frac{2k+1}{2L+1}\right],$$

where $x=x(\beta |\theta_{1},\theta_{2})$ is defined as follows:

(67) $$\cosh x=1+3\left(\beta_{c}/\beta -1\right)+\sum_{\nu =1}^{2}\left(1-\cos\theta_{\nu }\right).$$

The summations in Equation (66) can be performed using [65] the following identity:

(68) $$\frac{\cosh\left[(L+1/2)x\right]}{\cosh(x/2)}=\prod_{k=0}^{L-1}2\left[\cosh x-\cos\pi \frac{2k+1}{2L+1}\right].$$

With the help of the identity, one derives the following:

(69) $$S^{\left(\mathrm{ND}\right)}\left(x,L\right)=\frac{1}{L}\ln\frac{\cosh\left[(L+1/2)x\right]}{\cosh(x/2)}.$$

Obviously, $\lim_{L\to \infty }S^{\left(\mathrm{ND}\right)}\left(x,L\right)=x$. Thus, the excess free energy under Neumann–Dirichlet boundary conditions only depends on the interaction term as follows:

(70) $$\begin{array}{ccc} \beta \Delta f_{\mathrm{ex},3}^{\left(\mathrm{ND}\right)}\left(\beta ,h=0\right) & = & \frac{1}{2}\;L\;\left[U_{L,3}^{\left(\mathrm{ND}\right)}\left(\beta \right)-U_{\infty ,3}\left(\beta \right)\right]\\ & = & \frac{L}{8\pi^{2}}\;\int_{-\pi }^{\pi }d\theta_{1}\int_{-\pi }^{\pi }d\theta_{2}\;\left[\frac{1}{L}\ln\frac{\cosh\left[(L+1/2)x\right]}{\cosh(x/2)}-x\right]\\ & = & \frac{1}{8\pi^{2}}\;\int_{-\pi }^{\pi }d\theta_{1}\int_{-\pi }^{\pi }d\theta_{2}\;\left[\ln\left(\frac{e^{-(2L+1)x}+1}{e^{-x}+1}\right)\right]. \end{array}$$

Thus, $\beta \Delta f_{\mathrm{ex},3}^{\left(\mathrm{ND}\right)}\left(\beta ,h=0\right)$ can be decomposed into the sum of $g_{1}\left(L,\phi \right)$ and $g_{2}\left(L,\phi \right)$, where

(71) $$g_{1}\left(L,\beta \right)=\frac{1}{8\pi^{2}}\;\int_{-\pi }^{\pi }d\theta_{1}\int_{-\pi }^{\pi }d\theta_{2}\;\ln\left(e^{-(2L+1)x}+1\right),$$

and

(72) $$g_{2}\left(L,\beta \right)=-\frac{1}{8\pi^{2}}\;\int_{-\pi }^{\pi }d\theta_{1}\int_{-\pi }^{\pi }d\theta_{2}\;\ln\left(e^{-x}+1\right).$$

Let us consider the behavior of $g_{1}$ and $g_{2}$ in the scaling regime:

(73) $$x_{t}=6\left(\beta_{c}/\beta -1\right){\left(2L+1\right)}^{2}=O\left(1\right).$$

Let us first start with the function $g_{1}\left(L,\phi \right)$. Obviously, if x=O(1), then $g_{1}$ will be exponentially small. Thus, we must consider the regime (2L+1)x=O(1). It follows that x≪1. From Equation (67), we obtain the following:

(74) $$1+\frac{1}{2}x^{2}=3\left(\beta_{c}/\beta -1\right)+\frac{1}{2}\left(\theta_{1}^{2}+\theta_{2}^{2}\right).$$

Furthermore,

(75) $$x^{2}=6\left(\beta_{c}/\beta -1\right)+\left(\theta_{1}^{2}+\theta_{2}^{2}\right)=6\left(\beta_{c}/\beta -1\right)+r^{2},$$

where we have introduced polar coordinates. In terms of them, $g_{1}\left(L,\beta \right)$ becomes the following:

(76) $$\begin{array}{ccc} g_{1}\left(L,\beta \right) & ≃ & \frac{1}{4\pi }\int_{0}^{R}\ln\left(e^{-(2L+1)x}+1\right)dr^{2}≃\frac{1}{4\pi }\int_{\sqrt{6(\beta_{c}/\beta -1)}}^{\infty }\ln\left(e^{-(2L+1)x}+1\right)dx^{2}\\ & = & -\frac{1}{4\pi }\frac{\sqrt{x_{t}}\;{\mathrm{Li}}_{2}\left(-e^{-\sqrt{x_{t}}}\right)+{\mathrm{Li}}_{3}\left(-e^{-\sqrt{x_{t}}}\right)}{{\left(2L+1\right)}^{2}}, \end{array}$$

where *R* can be defined from the constraint $\left(2\pi \right)\times \left(2\pi \right)=4\pi^{2}=\pi R^{2}$, i.e., $R=2\sqrt{\pi }$.

Next, we deal with $g_{2}\left(L,\phi \right)$. Taking into account that $x_{L}$ is small, we derive the following:

(77) $$\begin{array}{ccc} g_{2}\left(L,\beta \right) & = & -\frac{1}{8\pi^{2}}\;\int_{-\pi }^{\pi }d\theta_{1}\int_{-\pi }^{\pi }d\theta_{2}\;\ln\left(e^{-x}+1\right)≃-\frac{1}{8\pi^{2}}\;\int_{-\pi }^{\pi }d\theta_{1}\int_{-\pi }^{\pi }d\theta_{2}\;\left[\ln2-\frac{1}{2}x\right]\\ & = & -\frac{1}{2}\ln2+\frac{1}{16\pi^{2}}\;\int_{-\pi }^{\pi }d\theta_{1}\int_{-\pi }^{\pi }d\theta_{2}\;x≃-\frac{1}{2}\ln2+\frac{1}{8\pi }\;\int_{\sqrt{6(\beta_{c}/\beta -1)}}^{R}xdx^{2}\\ & = & -\frac{1}{2}\ln2+\frac{1}{12\pi }\left\{R^{3}-{\left(\frac{\sqrt{x_{t}}}{2L+1}\right)}^{3}\right\}. \end{array}$$

Note that for $x_{t}=O\left(1\right)$, one knows that the *L*-dependent part $\Delta g_{2}\left(L,\beta \right)$ of $g_{2}$ has $\Delta g_{2}\left(L,\phi \right)\propto L^{-3}$, i.e., $\Delta g_{2}$ is one order of magnitude *smaller* than $g_{1}$. Because of this, $g_{2}$ only contributes sub-leading contributions to the *L*-dependent part of the excess free energy and, therefore, to the Casimir force. Based on the above, we are no longer interested in the $g_{2}$ function.

Summarizing the above, we conclude that the excess free energy can be written in the following scaling form:

(78) $$\beta \Delta f_{\mathrm{ex},3}^{\left(\mathrm{ND}\right)}\left(\beta ,h=0\right)=-\frac{1}{L^{2}}X_{\mathrm{ex}}\left(a_{\beta }tL^{1/\nu }\right),$$

where $a_{\beta }$ is a non-universal constant, and $X_{\mathrm{ex}}$ is a universal scaling function, $t=\left(T-T_{c}\right)/T_{c}$, where *T* is the temperature of the system, and $T_{c}$ is its bulk temperature. From Equation (76), taking into account that ν=1/2 has ${\left(2L+1\right)}^{2}≃4L^{2}≃L^{1/\nu }$, we identify the following:

(79) $$X_{\mathrm{ex}}\left(x_{t}\right)=\frac{1}{16\pi }\left[\sqrt{x_{t}}\;{\mathrm{Li}}_{2}\left(-e^{-\sqrt{x_{t}}}\right)+{\mathrm{Li}}_{3}\left(-e^{-\sqrt{x_{t}}}\right)\right].$$

#### 3.1.4. The Behavior of the Field Term $P_{\Lambda }^{\left(\tau \right)}\left(\beta ,h_{\Lambda }\right)$

The dependence of free energy on the field variable is provided by the “field term”, which presented in Equation (53). For an homogeneous field *h* and (per)≡(p,p,p) and ND≡(p,p,ND) boundary conditions, it is easy to obtain the following:

- For (p,p,p) boundary conditions,
  (80) $${\hat{h}}_{\Lambda }^{\left(\mathrm{per}\right)}\left(k\right)=\sum_{r\in \Lambda }h\left(r\right){\bar{u}}_{\Lambda }^{\left(\left(\mathrm{per}\right)\right)}\left(r,k\right)=\sqrt{L_{1}L_{2}L_{3}}\;\delta_{k_{1},0}\delta_{k_{2},0}\delta_{k_{3},0}\;h$$
  and
  (81) $$P_{L}^{\left(\mathrm{per}\right)}\left(K,h;\phi \right)=\frac{h^{2}}{6\beta (\beta_{c}/\beta -1)}.$$
  Obviously,
  (82) $$P_{\infty }\left(\beta ,h\right)=\lim_{L\to \infty }P_{L}^{\left(\mathrm{per}\right)}\left(K,h;\phi \right)=\frac{h^{2}}{6\beta (\beta_{c}/\beta -1)}.$$
- For (p,p,ND) boundary conditions,
  (83) $${\hat{h}}_{\Lambda }^{\left(\mathrm{ND}\right)}\left(k\right)=\sum_{r\in \Lambda }h\left(r\right){\bar{u}}_{\Lambda }^{\left(\mathrm{ND}\right)}\left(r,k\right)=2\sqrt{\frac{L_{1}L_{2}}{2L_{3}+1}}\;\delta_{k_{1},0}\delta_{k_{2},0}\;h\;\sum_{r=1}^{L_{3}}\cos\left[\left(r-1/2\right)\;\varphi_{L_{3}}^{\left(\mathrm{ND}\right)}\left(k_{3}\right)\right],\varphi_{L_{3}}^{\left(\mathrm{ND}\right)}\left(k_{3}\right)=\pi \frac{2k_{3}-1}{2L_{3}+1}.$$

Thus, setting $k_{3}=k,r_{3}=r$ and $L_{3}=L$ for a film geometry, we arrive at the following:

(84) $$P_{L}^{\left(\mathrm{ND}\right)}\left(\beta ,h\right)=\frac{4h^{2}}{\beta L(2L+1)}\sum_{k=1}^{L}\frac{{\left|\sum_{r=1}^{L}\cos\left[\left(r-1/2\right)\;\pi \frac{2k-1}{2L+1}\right]\right|}^{2}}{6\left(\beta_{c}/\beta -1\right)+2\left(1-\cos\pi \frac{2k-1}{2L+1}\right)}.$$

It is easy to show that

(85) $$2\sum_{r=1}^{L}\cos\left(\pi \left(r-1/2\right)\frac{\left(2k-1\right)}{\left(2L+1\right)}\right)=\frac{\sin\left(\pi \frac{2k-1}{2L+1}L\right)}{\sin\left(\frac{\pi }{2}\frac{2k-1}{2L+1}\right)}.$$

Thus, one has

(86) $$P_{L}^{\left(\mathrm{ND}\right)}\left(\beta ,h\right)=\frac{h^{2}}{\beta L(2L+1)}\sum_{k=1}^{L}\frac{\cot^{2}\left(\frac{\pi }{2}\frac{2k-1}{2L+1}\right)}{6\left(\beta_{c}/\beta -1\right)+2\left(1-\cos\pi \frac{2k-1}{2L+1}\right)}.$$

Let us consider the small *k* behavior of the above sum. Here, one derives the following:

(87) $$\begin{array}{ccc} P_{L}^{\left(\mathrm{ND}\right)}\left(\beta ,h\right) & ≃ & \frac{h^{2}}{\beta }\frac{1}{L(2L+1)}\sum_{k=1}^{L}\frac{1}{{\left[\frac{\pi (2k-1)}{2(2L+1)}\right]}^{2}\left\{6\left(\beta_{c}/\beta -1\right)+{\left[\frac{\pi (2k-1)}{2L+1}\right]}^{2}\right\}}\\ & ≃ & \frac{4}{\pi^{2}}\frac{h^{2}}{\beta }\frac{{\left(2L+1\right)}^{3}}{L}\sum_{k=1}^{L}\frac{1}{{\left(2k-1\right)}^{2}\left[x_{t}+\pi^{2}{\left(2k-1\right)}^{2}\right]}\\ & = & \frac{h^{2}}{\beta }\frac{{\left(2L+1\right)}^{3}}{L}\left\{\frac{1}{2x_{t}}\left[1-\frac{\tanh\left(\sqrt{x_{t}}/2\right)}{\sqrt{x_{t}}/2}\right]+O\left(L^{-3}\right)\right\}. \end{array}$$

In the limits $x_{t}\to 0$ and $x_{t}\to \infty$ for the behavior of the field term, one obtains the following:

(88) $$P_{L}^{\left(\mathrm{ND}\right)}\left(\beta ,h\right)≃\frac{h^{2}}{\beta }\frac{{\left(2L+1\right)}^{3}}{L}\left\{\begin{array}{cc} 1/24+O(x_{t}), & x_{t}\to 0;\\ \; & \\ 1/\left(2x_{t}\right)+O\left[\exp(-\sqrt{x_{t}})\right], & x_{t}\gg 1. \end{array}\right.$$

When L→∞, then $x_{t}\to \infty$, and we obtain the following:

(89) $$\lim_{L\to \infty }P_{L}^{\left(\mathrm{ND}\right)}\left(\beta ,h\right)=\frac{h^{2}}{6\beta (\beta_{c}/\beta -1)},$$

which indeed equals the bulk expression (see Equation (82)).

From Equation (87), for the behavior of the susceptibility in the finite system, we derive the following:

(90) $$\chi_{L}^{\left(\mathrm{ND}\right)}\left(\beta ,h\right)=\frac{1}{\beta }\frac{{\left(2L+1\right)}^{3}}{L}\frac{1}{x_{t}}\left[1-\frac{\tanh\left(\sqrt{x_{t}}/2\right)}{\sqrt{x_{t}}/2}\right].$$

According to the finite-size scaling theory [46,68],

(91) $$\chi_{L}^{\left(\zeta \right)}\left(t\right)=a_{h}L^{\gamma }X_{\chi }\left(a_{\beta }tL^{1/\nu }\right),$$

where $a_{h}$ and $a_{\beta }$ are non-universal constants, and $X_{\chi }$ is a universal scaling function, $t=\left(T-T_{c}\right)/T_{c}$, where *T* is the temperature of the system, and $T_{c}$ is its bulk temperature. From Equation (90), taking into account that ${\left(2L+1\right)}^{3}/L≃8L^{2}$, we identify the following:

(92) $$\gamma =2,\;\nu =1/2,\;\mathrm{and}\;tL^{2}=x_{t}.$$

It is clear that the field term in the free energy of the finite system will be of the same order as the field term, i.e., $\propto L^{-3}$ if $h\propto L^{-5/2}$. In order to achieve that, we define a field dependent scaling variable:

(93) $$x_{h}=\beta^{-1/2}{\left(2L+1\right)}^{3/2}L\;h.$$

In terms of the variable, Equation (87) becomes the following:

(94) $$P_{L}^{\left(\mathrm{ND}\right)}\left(x_{h},x_{t}\right)=\frac{x_{h}^{2}}{L^{3}}X_{\chi }\left(x_{t}\right),\;\mathrm{where}\;X_{\chi }\left(x_{t}\right)=\frac{1}{2x_{t}}\left[1-\frac{\tanh\left(\sqrt{x_{t}}/2\right)}{\sqrt{x_{t}}/2}\right].$$

**Figure 7:**
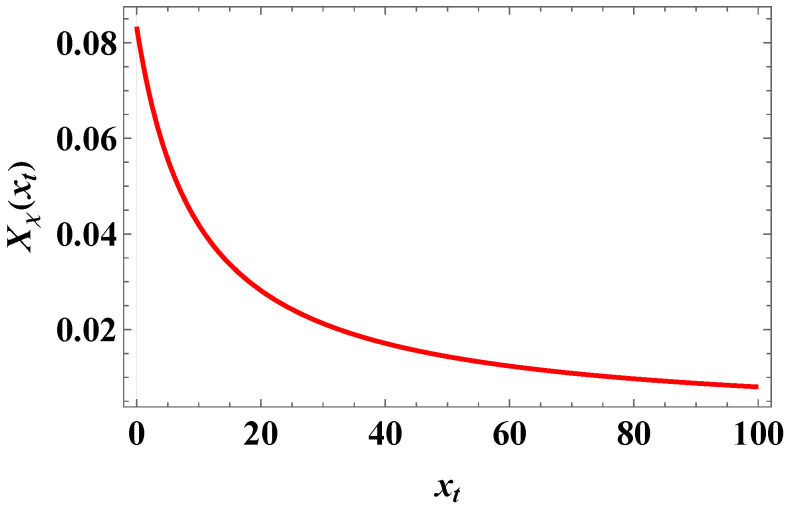
The behavior of the scaling function $X_{\chi }\left(x_{t}\right)$.

The behavior of the scaling function $X\chi (x_{t})$ is presented in Figure 7.

Then, for the excess free energy related to the field term (see Equation (51)), one derives the following:

(95) $$\beta \Delta f_{\mathrm{ex},3}^{\left(\mathrm{ND}\right)}\left(\beta ,h\right)=-\frac{1}{2}L\left[P_{L}^{\left(\mathrm{ND}\right)}\left(h;\beta \right)-P_{\infty }\left(h;\beta \right)\right]=\frac{x_{h}^{2}}{L^{2}}\frac{\tanh\left(\sqrt{x_{t}}/2\right)}{2x_{t}^{3/2}}.$$

### 3.2. The Behavior of the Casimir Force

Let us determine the contributions of the interaction term $\Delta F_{\mathrm{Cas}}^{\left(\mathrm{ND}\right)}\left(\beta ,h=0\right)$ and the field term $\Delta F_{\mathrm{Cas}}^{\left(\mathrm{ND}\right)}\left(\beta ,h\neq 0\right)$. Obviously, one has

(96) $$\Delta F_{\mathrm{Cas},3}^{\left(\mathrm{ND}\right)}\left(\beta ,h\right)=\Delta F_{\mathrm{Cas},3}^{\left(\mathrm{ND}\right)}\left(\beta ,h=0\right)+\Delta F_{\mathrm{Cas},3}^{\left(\mathrm{ND}\right)}\left(\beta ,h\neq 0\right).$$

We start by determining the behavior of $\Delta F_{\mathrm{Cas}}^{\left(\mathrm{ND}\right)}\left(\beta ,h=0\right)$. By definition, it is equal to the following:

(97) $$\Delta F_{\mathrm{Cas},3}^{\left(\mathrm{ND}\right)}\left(\beta ,h=0\right)\equiv -\frac{\partial }{\partial L}\beta \Delta f_{\mathrm{ex},3}^{\left(\mathrm{ND}\right)}\left(\beta ,h=0\right).$$

From Equation (70), we derive the *exact* expression as follows:

(98) $$\beta \Delta F_{\mathrm{Cas},3}^{\left(\mathrm{ND}\right)}\left(\beta ,h=0\right)=\frac{1}{4\pi^{2}}\;\int_{-\pi }^{\pi }d\theta_{1}\int_{-\pi }^{\pi }d\theta_{2}\;\frac{x}{e^{(2L+1)x}+1}.$$

Here, we do not make any assumption about *L*. Naturally, we will only obtain a scaling form of $\beta \Delta F_{\mathrm{Cas},3}^{\left(\mathrm{ND}\right)}\left(\beta ,h=0\right)$ for L≫1. Then, Equation (74) is valid and, after performing the integration, we arrive at the following:

(99) $$\begin{array}{ccc} \beta \Delta F_{\mathrm{Cas},3}^{\left(\mathrm{ND}\right)}\left(\beta ,h=0\right) & = & -\frac{1}{(2{\left(L+1\right)}^{3}}\frac{1}{\pi }\left\{{\mathrm{Li}}_{3}\left(-e^{-\sqrt{x_{t}}}\right)+\sqrt{x_{t}}{\mathrm{Li}}_{2}\left(-e^{-\sqrt{x_{t}}}\right)-\frac{1}{2}x_{t}\log\left(e^{-\sqrt{x_{t}}}+1\right)\right\}\\ & = & \frac{1}{{\left(L+1/2\right)}^{3}}X_{\mathrm{Cas},3}\left(x_{t}\right), \end{array}$$

where

(100) $$X_{\mathrm{Cas},3}\left(y\right)=-\frac{1}{8\pi }\left\{{\mathrm{Li}}_{3}\left(-e^{-\sqrt{x_{t}}}\right)+\sqrt{x_{t}}{\mathrm{Li}}_{2}\left(-e^{-\sqrt{x_{t}}}\right)-\frac{1}{2}x_{t}\log\left(e^{-\sqrt{x_{t}}}+1\right)\right\}.$$

**Figure 8:**
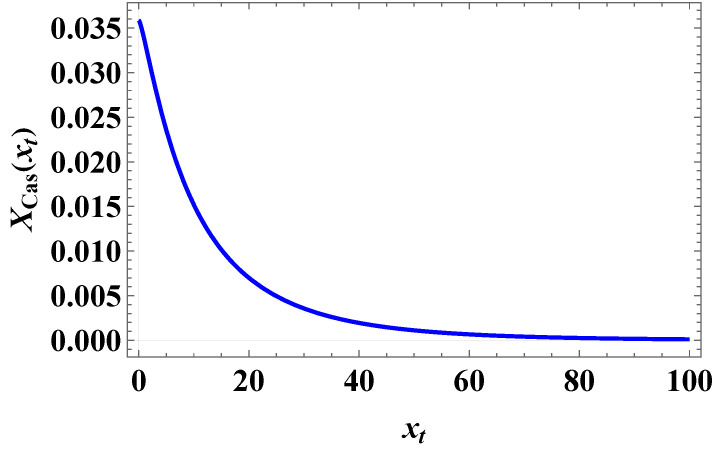
The behavior of the scaling function $X_{\mathrm{Cas}}\left(x_{t}\right)$ when h=0.

The behavior of the scaling function $X_{Cas}\left(x_{t},h=0\right)$ is provided in Figure 8. Obviously, the function is positive, which means that the Casimir force is repulsive when the external field is zero. For the Casimir amplitude, we obtain the following:

(101) $$\Delta_{\mathrm{Cas},3}^{\left(\mathrm{ND}\right)}\equiv X_{\mathrm{Cas},3}\left(x_{t}=0,h=0\right)/2=\frac{3}{64\pi }\zeta \left(3\right).$$

Obviously, Equation (101) coincides with the corresponding result for the Gaussian model obtained via studying the O(n), n=1,d=3$\Phi^{4}$ model (see ([26], Equation (6.99))). Analogically, after properly renaming the scaling variable, the expression Equation (100) of the scaling function of the force coincides with the corresponding one for the O(n), n=1,d=3$\Phi^{4}$ model (see ([26], Equation (6.104))).

Let us now determine the *h*-dependent part of the Casimir force. By definition, one has the following:

(102) $$\Delta F_{\mathrm{Cas},3}^{\left(\mathrm{ND}\right)}\left(\beta ,h\right)\equiv -\frac{\partial }{\partial L}\beta \Delta f_{\mathrm{ex},3}^{\left(\mathrm{ND}\right)}\left(\beta ,h\right).$$

Then, from Equation (95), one obtains

(103) $$\begin{array}{ccc} \Delta F_{\mathrm{Cas},3}^{\left(\mathrm{ND}\right)}\left(\beta ,h\right) & = & -\frac{\partial }{\partial L}\left[\frac{x_{h}^{2}}{L^{2}}\frac{\tanh\left(\sqrt{x_{t}}/2\right)}{2x_{t}^{3/2}}\right]≃-\frac{x_{h}^{2}}{2L^{2}\left(1+2L\right)}\left[\frac{{\sec h}^{2}\left(\sqrt{x_{t}}/2\right)}{x_{t}}\right] \end{array}$$

(104) $$\begin{array}{ccc} & = & \frac{1}{L^{2}\left(L+1/2\right)}X_{\mathrm{Cas},3}\left(x_{t},x_{h}\right), \end{array}$$

where

(105) $$X_{\mathrm{Cas},3}\left(x_{t},x_{h}\right)=-\frac{x_{h}^{2}}{4}\left[\frac{{\sec h}^{2}\left(\sqrt{x_{t}}/2\right)}{x_{t}}\right]<0.$$

**Figure 9:**
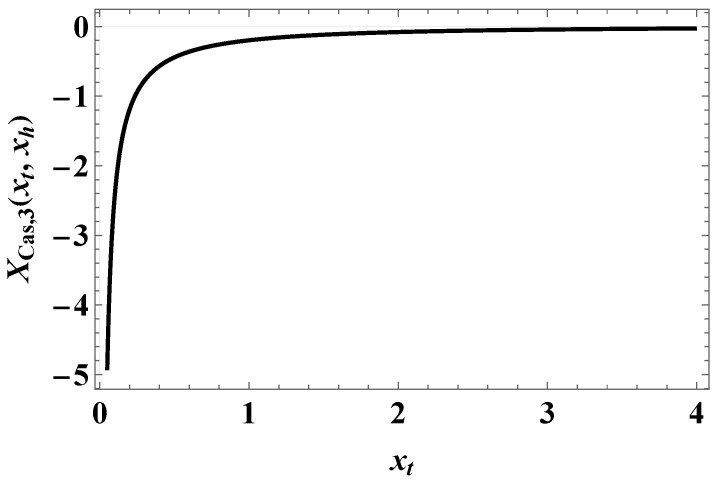
The behavior of the scaling function $X_{\mathrm{Cas}}\left(x_{t},x_{h}=1\right)$. We observe that the force is *attractive*.

A visualization of $X_{\mathrm{Cas},3}\left(y,x_{h}\right)$ as a function of *y* for $x_{h}=1$ is shown in Figure 9.

**Figure 10:**
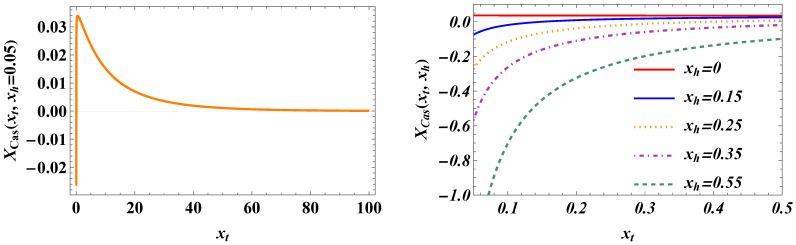
The behavior of the scaling function of the total Casimir force as a function of *y* for several values of $x_{h}$. (**Left panel**): We see that for $x_{h}=0.05$, the force is attractive very near the critical temperature, then becomes repulsive with an increase in $x_{t}$ (i.e., of *T*). (**Right panel**): It is clear that for the zero field, the force is repulsive. Then, for small values of $x_{h}$, the force changes from attractive to repulsive with the increase in $x_{t}$ (i.e., of the temperature), while for large values of $x_{h}$, the force becomes attractive for all values of *T* (i.e., $x_{t}$).

The total Casimir force is the sum of $X_{\mathrm{Cas},3}\left(x_{t}\right)$ (see Equation (100)) and $X_{\mathrm{Cas},3}\left(x_{t},x_{h}\right)$, as given by Equation (105). The plot of the result as a function of $x_{t}$ for $x_{h}=0.05$ is shown in Figure 10. As we can see, the force can be both positive and negative, i.e., *repulsive* and *attractive*.

**Figure 11:**
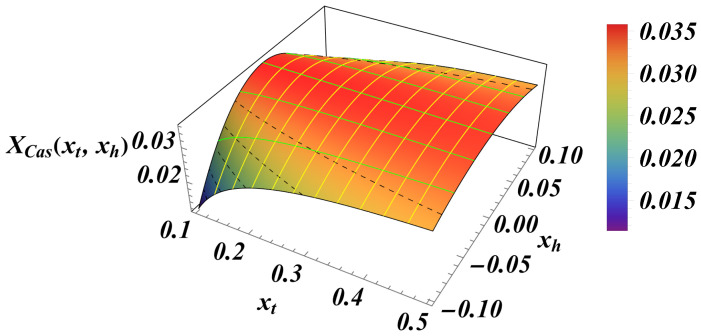
The behavior of the scaling function $X_{\mathrm{Cas}}\left(x_{t},x_{h}\right)$. Here, $x_{t}\in \left[0.1,0.5\right]$ and $x_{h}\in \left[-0.1,0.1\right]$.

The overall 3D behavior of the force as a function of both $x_{t}$ and $x_{h}$ is presented in Figure 11.

## 4. The Casimir Force Within the Mean-Field Model

We start by defining the mean-field model used in the current study.

### 4.1. The Ginzburg–Landau Functional

In the present work, we consider the standard $\phi^{4}$ Ginzburg–Landau functional:

(106) $$F\left[\phi |\tau ,h,L\right]=\int_{0}^{L}L(\phi ,\phi^{\prime }|\tau ,h)\;dz,$$

with

(107) $$L\left(\phi ,\phi^{\prime }|\tau ,h\right)=\frac{1}{2}\phi^{\prime 2}+\frac{1}{2}\tau \phi^{2}+\frac{1}{4}g\phi^{4}-h\phi .$$

Here, $L,\;g\in R^{+}$, while τ,h∈R, z∈(0,L), and ϕ=ϕ(z) are the independent and dependent variables, respectively, and the prime indicates differentiation with respect to the *z* variable.

The functional (106) describes a critical system of the Ising type in a film geometry $\infty^{2}\times L$, where the film thickness *L* is supposed to be along the *z* axis. In Equation (106), ϕ(z|τ,h,L) is the order parameter of the system, which is assumed to depend on the perpendicular position z∈(0,L) only, *g* is the bare coupling constant, $\tau =\left(T-T_{c}\right)/T_{c}$ is the bare reduced temperature, and *h* is the external ordering field. Given τ, *h*, and *L*, the physical state of the regarded system is described by the minimizer of the respective Ginzburg–Landau functional F[ϕ;τ,h,L] given above whose extremals are determined by the solutions of the corresponding Euler–Lagrange equation:

(108) $$\frac{d}{dz}\frac{\partial L}{\partial \phi^{\prime }}-\frac{\partial L}{\partial \phi }=0.$$

In case the of Lagrangian density, L is defined by Equation (107), and Equation (108) reads as follows:

(109) $$\phi^{\prime \prime }-\phi \left[\tau +g\;\phi^{2}\right]+h=0.$$

Multiplying Equation (109) by $\phi^{\prime }$ and integrating once over *z*, one obtains the following:

(110) $$P\left[\phi \right]\equiv \frac{1}{2}{\phi^{\prime }}^{2}-\frac{1}{2}\tau \phi^{2}-\frac{1}{4}g\phi^{4}+h\phi ,$$

which is the first integral of Equation (109), cf., e.g., [26]. This means that *P* is a constant on any smooth solution ϕ(z|τ,h,L) of the Euler–Lagrange Equation (109).

**Figure 12:**
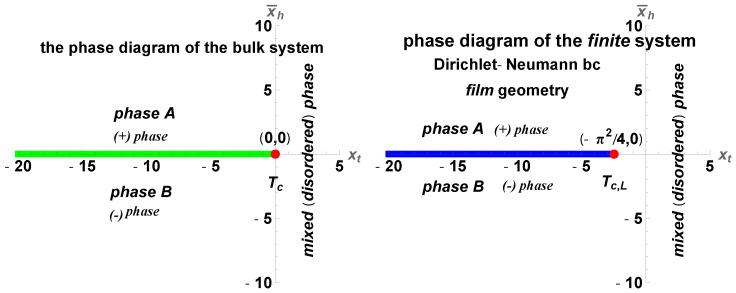
Phase diagrams. (**Left panel**): The phase diagram of the bulk system. (**Right panel**): The phase diagram of the finite system with Dirichlet–Neumann boundary conditions. In the bulk system, a phase transition of the first order occurs when crossing the phase coexistence line that is at ${\bar{x}}_{h}=0$ and spans for $T\in (0,T=T_{c})$. At $T=T_{c}$, the system exhibits second-order phase transition. In the finite system, the coexistence line is at ${\bar{x}}_{h}=0$ and spans for $T\in (0,T=T_{c,L})$. Second-order phase transition occurs at $T=T_{c,L}\equiv \left(-\pi^{2}/4,0\right)$. Note the change with Dirichlet–Dirichlet boundary conditions, where the critical point is at $T_{c,L}=\left(-\pi^{2},0\right)$.

The phase diagram of the finite system with Dirichlet–Neumann boundary conditions is shown in Figure 12.

In general, the thermodynamic Casimir force $F_{\mathrm{Cas}}\left(\tau ,h,L\right)$ in such a system is the *excess pressure* over the bulk pressure, acting on the boundaries of the finite system, which is due to the finite size of that system, i.e.,

(111) $$F_{\mathrm{Cas}}\left(\tau ,h,L\right)=P_{L}\left(\tau ,h\right)-P_{b}\left(\tau ,h\right).$$

Here, $P_{L}$ is the pressure in the finite system, while $P_{b}$ is the pressure in the infinite system.

Now, assuming that the thickness *L* of the film is free to move, the variation δF of the free energy F[ϕ|τ,h,L] of the finite system is provided as follows:

(112) $$\delta F=\int_{0}^{L}\left(\frac{\partial L}{\partial \phi }-\frac{d}{dz}\frac{\partial L}{\partial \phi^{\prime }}\right)\delta \phi dz+L_{\phi^{\prime }}\delta \phi {|}_{0}^{L}-\left(\phi^{\prime }L_{\phi^{\prime }}-L\right)\delta z{|}_{0}^{L}$$

(see ([69], p. 54), ([70], p. 260), and [71]), where δz and δϕ are the variations in the independent and dependent variables, while

(113) $$T_{zz}=\phi^{\prime }L_{\phi^{\prime }}-L$$

is the one-dimensional counterpart of the stress tensor (see, [72,73]). Relation (112) estimates the change in the finite-size contribution to the free energy of the system corresponding to a small variation in the variables, including the variation in the film thickness *L*. In this sense, ${\left.T_{zz}\right|}_{L}$ can be interpreted (see [63,74]) as the pressure in the finite system:

(114) $$P_{L}={\left.T_{zz}\right|}_{L}.$$

On the other hand, taking into account Equations (107) and (113), one can see that

(115) $$T_{zz}=P\left[\phi \right].$$

Hence, $T_{zz}$ is a constant on any smooth solution ϕ(z|τ,h,L) of the Euler–Lagrange Equation (109), including the minimizer of the Ginzburg–Landau functional (106). Thus, the pressure in the finite system is as follows:

(116) $$P_{L}\left(\tau ,h\right)=\frac{1}{2}{\phi_{min}^{\prime }}^{2}-\frac{1}{4}g\phi_{min}^{4}-\frac{1}{2}\tau \phi_{min}^{2}+h\phi_{min},$$

where $\phi_{min}$ is the foregoing minimizer.

As for the bulk system, it is easy to see following way of reasoning: corresponding pressure is

(117) $$P_{b}\left(\tau ,h\right)=-\frac{1}{4}g\phi_{b}^{4}-\frac{1}{2}\tau \phi_{b}^{2}+h\phi_{b}.$$

Here, the value $\phi_{b}$ of the order parameter of the bulk system is determined as the constant solution of Equation (109), i.e., the root of the cubic equation:

(118) $$-\phi_{b}\left[\tau +g\;\phi_{b}^{2}\right]+h=0,$$

which minimizes

(119) $$L_{b}=\frac{1}{2}\tau \phi_{b}^{2}+\frac{1}{4}g\phi_{b}^{4}-h\phi_{b}.$$

Of course, $\phi_{b}$ does not depend on the boundary conditions at all. Let us note that $P_{b}=-L_{b}$, i.e., $P_{b}$, has its *maximum* over the solution $\phi_{b}$ of the cubic equation for (118).

Obviously, the relation (116) does *not* depend on the boundary conditions applied to the finite system, too. This dependence arises solely from the dependency of the order parameter profile that minimizes the particular boundary value problem considered.

In light of the above, it is evident that once the order parameter profile $\phi_{min}$ and its bulk value $\phi_{b}$ are known in analytic form for given values of the parameters τ and *h*, then the respective Casimir force is determined in an exact manner by Equation (111).

In the current article, we consider the Dirichlet–Neumann boundary conditions:

(120) $$\phi \left(z=0|\tau ,h,L\right)=0\;and\;\frac{\partial }{\partial z}\phi {\left(z|\tau ,h,L\right)}_{{|}_{z=1}}=0.$$

In addition, ν is a critical exponent characterizing the behavior of the correlation length, while Δ is another exponent related to the behavior of, say, order parameter as a function of the external field *h*.

It is convenient to introduce the following new parameters:

(121) $$x_{t}=\frac{\tau L^{1/\nu }}{{\left[\xi_{0}^{+}\right]}^{1/\nu }},\;x_{h}=\frac{\sqrt{2g}hL^{\Delta /\nu }}{{\left[\xi_{0,h}\right]}^{\Delta /\nu }},$$

including variables

(122) $$\zeta =z/L,\;\phi \left(z\right)=\sqrt{\frac{2}{g}}\;L^{-\beta /\nu }X_{m}\left(\zeta |x_{t},x_{h}\right),$$

where β=ν=1/2 and Δ=3/2, while $\xi_{0}^{+}$ and $\xi_{0,h}$ are the respective amplitudes of the correlation length along the τ and *h* axes (see [26]). In terms of these new parameters and variables, Equations (106), (107), (109) and (110) become the following:

(123) $$F\left[X_{m}|x_{t},x_{h}\right]=\frac{1}{gL^{4}}\int_{0}^{1}L\left[X_{m},X_{m}^{\prime }|x_{t},x_{h}\right]d\zeta ,$$

(124) $$L\left[X_{m},X_{m}^{\prime }|x_{t},x_{h}\right]=X_{m}^{\prime 2}\left(\zeta \right)+X_{m}^{4}\left(\zeta \right)+x_{t}X_{m}^{2}\left(\zeta \right)-x_{h}X_{m}\left(\zeta \right),$$

(125) $$X_{m}^{\prime \prime }\left(\zeta \right)=X_{m}\left(\zeta \right)\left[x_{t}+2X_{m}^{2}\left(\zeta \right)\right]-\frac{x_{h}}{2},$$

and

(126) $$P\left[X_{m}\left(\zeta \right)\right]=X_{m}^{\prime 2}\left(\zeta \right)-X_{m}^{4}\left(\zeta \right)-x_{t}X_{m}^{2}\left(\zeta \right)+x_{h}X_{m}\left(\zeta \right),$$

respectively. The primes here and hereafter indicate differentiation with respect to the variable ζ∈[0,1]. Then, according to Equations (111), (116) and (117), the expression for the Casimir force $X_{\mathrm{Cas}}\left(x_{t},x_{h}\right)$ written by means of the new parameters (121) and variables (122) reads as follows:

(127) $$X_{\mathrm{Cas}}\left(x_{t},x_{h}\right)={\hat{X}}_{m}^{\prime 2}-\left({\hat{X}}_{m}^{4}-X_{b}^{4}\right)-x_{t}\left({\hat{X}}_{m}^{2}-X_{b}^{2}\right)+x_{h}\left({\hat{X}}_{m}-X_{b}\right),$$

where ${\hat{X}}_{m}$ and $X_{b}$ are the minimizers of the functional (123) and its “bulk counterpart” corresponding to $x_{t}$ and $x_{h}$.

As mentioned above, in the present article, we assume that the system is subject to Dirichlet–Neumann boundary conditions; that is,

(128) $$X_{m}\left(\zeta =0|x_{t},x_{h}\right)=0\;\mathrm{and}\;X_{m}^{\prime }\left(\zeta =1|x_{t},x_{h}\right)=0.$$

In other words, we are interested in the solution of Equation (125) that meets the conditions (128). It should be remarked that exact results associated with the Casimir effect have been derived in the cases of (+,+), (+,−), and Dirichlet–Dirichlet boundary conditions (see [26] for a review).

### 4.2. The Casimir Force for the Zero External Field

In [75], it was shown that for $x_{t}\in \left(-\infty ,-\pi^{2}/4\right]$ there are two order parameter profiles that minimize the functional (123) in the case of Dirichlet–Neumann boundary conditions and zero external fields. They can be expressed using an auxiliary parameter k∈[0,1] as follows:

(129) $${\hat{X}}_{m}\left(\zeta \right)=\pm \;k\;K\left(k\right)\mathrm{sn}\left(\zeta K\left(k\right)|k\right)$$

at

(130) $$x_{t}=-\left(k^{2}+1\right)K{\left(k\right)}^{2},$$

where K(·) is the complete elliptic integral of the first kind, and $\mathrm{sn}\left(\cdot |\cdot \right)$ is the sine Jacobi elliptic function. Simultaneously, it is easy to see that in this case,

(131) $$X_{b}=\frac{1}{\sqrt{2}}\sqrt{\left(k^{2}+1\right)K{\left(k\right)}^{2}}.$$

Now, substituting Equations (129) and (131) into Equation (127), one obtains the following:

(132) $$X_{\mathrm{Cas}}\left(x_{t},x_{h}=0\right)=-\frac{1}{4}{\left(k^{2}-1\right)}^{2}K{\left(k\right)}^{4}$$

for the Casimir force at $x_{t}\in \left(-\infty ,-\pi^{2}/4\right]$, as shown in Equation (130).

If $x_{t}\in \left(-\pi^{2}/4,0\right]$, then ${\hat{X}}_{m}=0$, $X_{b}=-\sqrt{-x_{t}/2}$, and, hence, according to Equation (127), the expression for the Casimir force reads as follows:

(133) $$X_{\mathrm{Cas}}\left(x_{t},x_{h}=0\right)=-\frac{x_{t}^{2}}{4}.$$

Finally, if $x_{t}\in \left(0,\infty \right)$, then $X_{\mathrm{Cas}}\left(x_{t},x_{h}=0\right)=0$. Combining these results, one can write down the following:

(134) $$\begin{array}{c} X_{\mathrm{Cas}}\left(x_{t},x_{h}=0\right)=\left\{\begin{array}{cc} -\frac{1}{4}{\left(k^{2}-1\right)}^{2}K{\left(k\right)}^{4}, & x_{t}\in \left(-\infty ,-\frac{\pi^{2}}{4}\right],\\ -x_{t}^{2}/4, & x_{t}\in \left(-\frac{\pi^{2}}{4},0\right],\\ 0, & x_{t}\in \left(0,\infty \right). \end{array}\right. \end{array}$$

**Figure 13:**
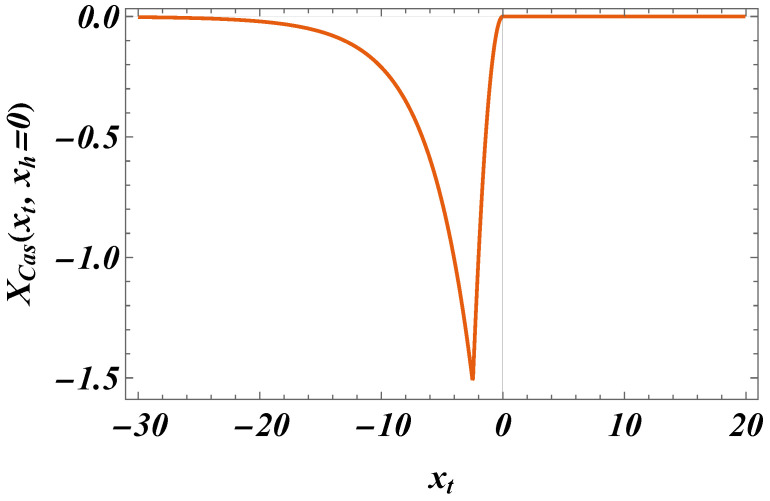
The behavior of the scaling function $X_{\mathrm{Cas}}\left(x_{t},x_{h}=0\right)$ for $x_{t}\in \left[-30,20\right]$. We observe that the force is *attractive*, contrary to the corresponding result for the Gaussian model.

The behavior of the scaling function $X_{\mathrm{Cas}}\left(x_{t},x_{h}=0\right)$ for $x_{t}\in \left[-30,30\right]$ is depicted in Figure 13.

### 4.3. The Casimir Force for the Non-Zero External Field

In [75], it was shown, following ([76], p. 454), that each solution of Equation (125) that meets the Dirichlet–Neumann boundary conditions can be written in the following form:

(135) $$X_{m}\left(\zeta |x_{t},x_{h},X_{m,r}\right)=X_{m,r}+\frac{6X_{m,r}\left(x_{t}+2X_{m,r}^{2}\right)-3x_{h}}{12℘\left(\zeta -1;g_{2},g_{3}\right)-\left(x_{t}+6X_{m,r}^{2}\right)},$$

where $X_{m,r}=X_{m,r}\left(x_{t},x_{h}\right)$ is a real number that depends only on the values of parameters $x_{t}$ and $x_{t}$. Here, $℘\left(\nu ;g_{2},g_{3}\right)$ is the Weierstrass elliptic function corresponding to the invariants $g_{2}$ and $g_{3}$ provided as follows:

(136) $$\begin{array}{ccc} & & g_{2}=\frac{1}{12}x_{t}^{2}-X_{m,r}\left(X_{m,r}^{3}+x_{t}X_{m,r}-x_{h}\right),\\ & & g_{3}=-\frac{1}{432}\left[27x_{h}^{2}+2x_{t}^{3}+72x_{t}X_{m,r}\left(X_{m,r}^{3}+x_{t}X_{m,r}-x_{h}\right)\right]. \end{array}$$

It is easy to see that $X_{m,r}$ is the value of the order parameter at the right end of the system since $℘\left(\nu ;g_{2},g_{3}\right)$ tends toward infinity when ν tends toward zero. It is also easy to see that $X_{m}^{\prime }\left(\zeta \to 1|x_{t},{\bar{x}}_{h},X_{m,r}\right)=0$, i.e., each function of the form (135) meets the boundary condition imposed on the right end of the system. The only remaining requirement is that $X_{m}\left(\zeta \to 0|x_{t},x_{h},X_{m,r}\right)=0$ leads to a transcendental equation from where we must determine $X_{m,r}$. Usually, one obtains several solutions to this equation. However, the one that corresponds to the physical reality is the one that minimizes the energy provided by Equations (123) and (124). In this way, we determine the order parameter profile ${\hat{X}}_{m}$ as a function of parameters $x_{t}$ and $x_{h}$. We also obtain $X_{b}$ as a function of $x_{t}$ and $x_{h}$

Finally, using Equation (127), we obtain the Casimir force $X_{\mathrm{Cas}}\left(x_{t},x_{h}\right)$.

## 5. Conclusions

As reported above, we obtained exact results for the Casimir force in two basic statistical mechanical models: the Gaussian and mean-field models. In the case of the Gaussian model, we performed the calculations for two realizations: a continuum version (see Section 2) and a lattice version (see Section 3 realizations). The mean-field model was considered in Section 4. The models were considered under Neumann–Dirichlet boundary conditions in the presence of an external magnetic field *h*.

**Figure 14:**
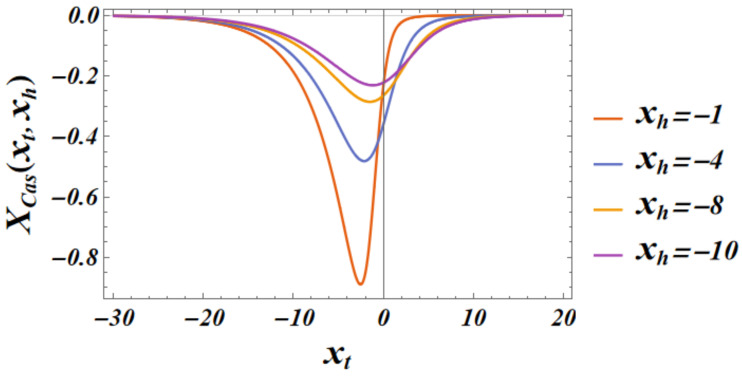
The behavior of the scaling function $X_{\mathrm{Cas}}\left(x_{t},x_{h}\right)$, $x_{t}\in \left[-30,20\right]$ for several values of $x_{h}$. We observe that the force is *attractive*.

**Figure 15:**
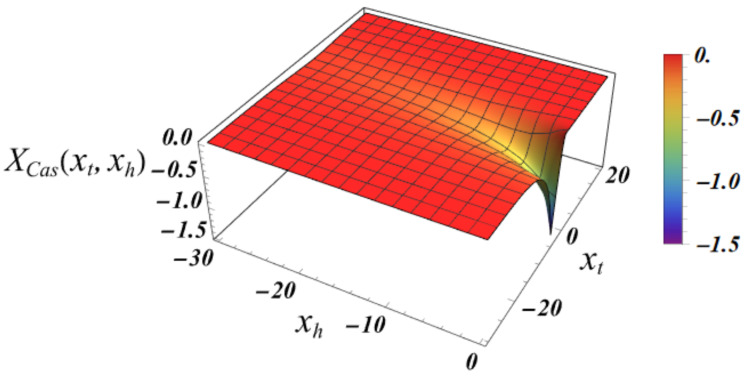
The behavior of the scaling function $X_{\mathrm{Cas}}\left(x_{t},x_{h}\right)$, $x_{t}\in \left[-30,20\right]$, $x_{h}\in \left[-30,0\right]$. We observe that the force is *attractive*.

We summarize our main results as follows:

(I) We derived exact closed-form expression for the free energy of the Gaussian model in both the continuum version (CGM) and the lattice formulation of the model (LGM). The results for the Casimir force can be written as a sum of the following:
  (i) Expressions pertinent to the h=0 case (see Equation (33) for CGM and Equation (100) for the LGM).
  (ii) Equations for the field-dependent parts of the force (see Equation (41) for the CGM, and Equation (105) for the LGM).
  We observe that these expressions are identical, as is to be expected on the ground of the universality hypothesis, provided proper definitions of the scaling variables are used.
(II) The behavior of the Casimir force in the CGM is shown in Figure 3 and Figure 5, and the behavior of the LGM is shown in Figure 8, Figure 9, Figure 10 and Figure 11. We observe that for h=0, the force is repulsive and, depending on the magnitude of *h*, it can be both repulsive or attractive for h≠0. Contrary to this behavior, we observe that the force in the MFM is *always* attractive, both for h=0 (see Figure 13) and h≠0 (see Figure 14 and Figure 15).

From all of the above, one can, at the very least, conclude the following:

• The sign of the Casimir force for the GM is not necessarily the same for h=0, for which case it is very well known (see [26,46,49,77]), as it is similar to that of h≠0.
• The predictions of the “workhorse” of statistical mechanics, i.e., the mean-field approach, particularly in studies of the Casimir force, can be wrong even with respect to the predicted sign of the force.

The results presented in the current article are based on exact analytical expressions for both the Gaussian and mean-field models.

As far as the thermodynamic Casimir effect has been investigated, most results pertain to a classical system in the grand canonical ensemble. It is, however, possible to consider ensemble-dependent fluctuation-induced forces as in [28,75,78,79], where it has been shown that these forces have behaviors that are quite different from those of the Casimir force under the same boundary conditions and with the same geometry. We note that all of the issues studied for Casimir forces could also be objects of investigation in, say, canonical or micro-canonical ensembles. We plan to extend the results reported in the current article to such ensembles. Let us note that in [26], via exact results for the one-dimensional Ising model in a fixed-order parameter *M* ensemble, we have shown that the fluctuation-induced force pertinent to this ensemble, which we termed the Helmholtz force, shows behavior similar to that appearing in certain versions of the Big Bang theory, e.g., strong repulsion at high temperatures, transitioning to moderate attraction for intermediate values of the temperature and then back to repulsion, albeit much more weakly than during the initial period of the highest temperature. It would be very interesting to check to which extent this result persists in other, somewhat more realistic, models like the ones considered in the current work. We stress that in customarily considered applications involving, say, the equilibrium Ising model with respect to binary alloys or binary liquids, if one insists on full rigor, the case with a fixed-order parameter must be addressed.

## Data Availability

Data is contained within the article.

## Abbreviations

The following abbreviations are used in this manuscript:

CCF	critical Casimir force
BC’s	boundary conditions
DN	Dirichlet–Neumann
GCE	grand canonical ensemble
LGM	lattice Gaussian model
CGM	continuum Gaussian model
